# Harnessing copper oxide nanoparticles for advanced photocatalytic, antimicrobial, and larvicidal applications

**DOI:** 10.1038/s41598-025-25441-2

**Published:** 2025-11-18

**Authors:** Ahmed M. El-Khawaga, Doaa S. R. Khafaga, Yosri A. Fahim, A. M. Fadl

**Affiliations:** 1 Department of Basic Medical Sciences, Faculty of Medicine, Galala University, New Galala City, 43511 Suez Egypt; 2 Department of Basic Medical Sciences, Health Sector, Galala University, New Galala City, 43511 Suez Egypt; 3 Biological Application Department, Nuclear Research Center, Egyptian Atomic Energy Authority, Cairo, Egypt

**Keywords:** Copper oxide, Photocatalysis, Antibacterial agent, *Culex pipiens*, Pollution remediation, Nanoscience and technology

## Abstract

Copper oxide nanoparticles (CuO-NPs) have garnered significant attention due to their multifunctional properties and diverse application areas. The synthesized CuO-NPs were characterized using XRD, SEM, HRTEM, and FTIR. The photocatalytic properties of CuO-NPs were assessed using methylene blue dye degradation under UV light. Various parameters such as pH, initial concentration of MB, and catalyst dose were investigated to determine their effects on photocatalytic efficiency. Kinetic analysis revealed that the degradation process followed a pseudo-first-order model. The antimicrobial efficacy of CuO-NPs was evaluated against Gram-positive and Gram-negative bacteria, demonstrating significant activity and providing insights into the underlying antibacterial mechanisms. Additionally, the toxicity of CuO-NPs was tested on *Culex pipiens* larvae, showing increased mortality rates with higher nanoparticle concentrations. Copper oxide nanoparticles elucidated the larvicidal effect on *Culex pipiens*. The lethal concentration (LC_50_) values were determined as 37.61 mg/L for 3rd instar larvae and 8.31 mg/L for 4th instar larvae. The study provides a comprehensive analysis of CuO-NPs’ potential in photocatalysis, antimicrobial applications, and insecticidal properties, contributing to the understanding of their multifunctional roles in various environmental and biological contexts.

## Introduction

CuO-NPs are highly significant in the field of environmental remediation and biopharmaceutical industries due to their noteworthy contributions^1^. CuO-NPs, which are a form of narrowband semiconductor with P-type conductivity, have important characteristics that greatly enhance their efficiency in solar applications and their ability to catalyze textile and cationic dyes^2^. The synthesized CuO-NPs demonstrated substantial degradation activity against several cationic dyes, such as crystal violet, methyl red, and methylene blue^3^. This study utilized methylene blue as a method to evaluate the degradative capability of CuO-NPS. Methylene blue (MB) is a cationic dye that has been extensively used in both biological research and the textile industry^4^. CuO-NPs are used as a powerful antibacterial agent against pathogenic bacterial strains^5^. There are multiple techniques for producing CuO-NPs, one of which being microwave-assisted procedures^6^, hydrothermal^7^, reverse micelles process^8^, and photochemical synthesis^9^. During the process of precipitating a solid phase from a solution, impurities that are usually soluble in the solution can adhere to the nuclei or crystals and be removed together with the primary solid as a single phase^10^. The phenomenon is known as co-precipitation. Co-precipitation refers to the process in which usually soluble compounds are carried out of a solution by a precipitate^11^. There are four distinct forms of co-precipitation: surface adsorption, mixed-crystal formation, occlusion, and mechanical entrapment. The synthesis of nanoparticles utilizing this approach is both convenient and economically efficient^12^. The nucleation process is a critical feature of co-precipitation, as it signifies the beginning of particle formation. Afterwards, other processes such as Ostwald ripening and aggregation occur^13^. These secondary processes determine the size, shape, and properties of particles. The reaction conditions, such as the rate of reactant addition and the stirring strength, have an impact on the particle size distribution, morphology, and particle sizes^14^. An important advantage of the co-precipitation technique is its simplicity^15^. The amalgamation of the constituents via chemical co-precipitation yields an end product with nearly impeccable stoichiometry, obviating the requirement for elevated temperature procedures. At times, the firing temperature may drop to a minimum of 800 ˚C^16,17^. The incidence of antibiotic resistance is rapidly increasing across many bacterial species, presenting a substantial concern in both clinical and public health sectors globally^18^. *Culex pipiens* is a vector for globally prevalent diseases such as Rift Valley fever and West Nile virus^19,20^, Filariasis^21^ and Denge fever^22^. Therefore, it is crucial to utilize other strategies for controlling *Cx. pipiens*. CuO-NPs have lately garnered considerable attention in several application domains and are currently undergoing intense research and development. The novelty of this work lies in the integration of photocatalytic, antimicrobial, and larvicidal properties within a single nanomaterial platform. These findings contribute to the growing field of nanotechnology-based solutions for environmental remediation, and public health. The central objective of this research is to evaluate the effectiveness of the synthesized CuO-NPs in three critical domains: (1) photocatalytic degradation of methylene blue dye under UV light, (2) antimicrobial activity against Gram-positive and Gram-negative bacteria, and (3) larvicidal toxicity against *Culex pipiens* larvae. By integrating these assessments, this study seeks to demonstrate the potential of CuO-NPs as a versatile nanomaterial for environmental remediation and vector control, while laying a foundation for their safe and scalable application.

## Materials and methods

### Chemical reagents

The chemicals used in the experiment were copper (II) chloride dihydrate, sodium hydroxide pellets, and methylene blue dye (MB, ≥ 97%), all of which were obtained from E-Merck Products. The experiment utilized analytical reagent-grade chemicals, which were employed without any additional purification. The experiment utilized distilled water and deionized water for the preparation of solutions and for cleaning purposes.

### Synthesis of CuO-NPs

CuO-NPs were synthesized via the precipitation method. 9.0 g of copper (II) chloride dihydrate and 5.4 g of sodium hydroxide pellets were dissolved separately in ethanol. The amount of ethanol used was the minimum required to fully dissolve copper (II) chloride dihydrate and sodium hydroxide separately. The experiment was incrementally adding sodium hydroxide solution to copper (II) chloride dihydrate solution while continuously stirring at room temperature. The solution exhibited a chromatic transition, starting from a green hue and gradually shifting towards a bluish-green shade, ultimately culminating in a black coloration throughout the course of the reaction. The resulting precipitate was identified as copper hydroxide. The precipitate was filtered and then washed with ethanol and deionized water. Afterwards, the solid was dried at around 50 °C in the dryer. In order to obtain crystalline CuO-NPs, the dried sample was annealed at a temperature of 600 °C. Afterwards, the annealed sample was crushed to get the powdered nanoparticles. The power sample was used to analyze and describe the characteristics of CuO-NPs^23^.

### Characterization of the CuO-NPs

Diverse characterization approaches were utilized to identify the synthesized CuO-NPs. The phase present in the synthesized sample was determined and examined using an X-ray diffraction (XRD) X’Pert PRO model, equipped with Cu Kα radiation (λ = 1.5418 Å). The morphological and semi-quantitative elemental analysis was conducted using an FEI Czech-type scanning electron microscope (SEM) operating at a voltage of 25–30 kilovolts (kv). The microscope had a magnification of 150X and an average working distance of 15 ml. A JEM-2100 model High Resolution Transmission Electron Microscope (HRTEM), produced by JEOL in Japan, was used for thorough morphological investigation.

### Photocatalytic setup

**Fig. 1:**
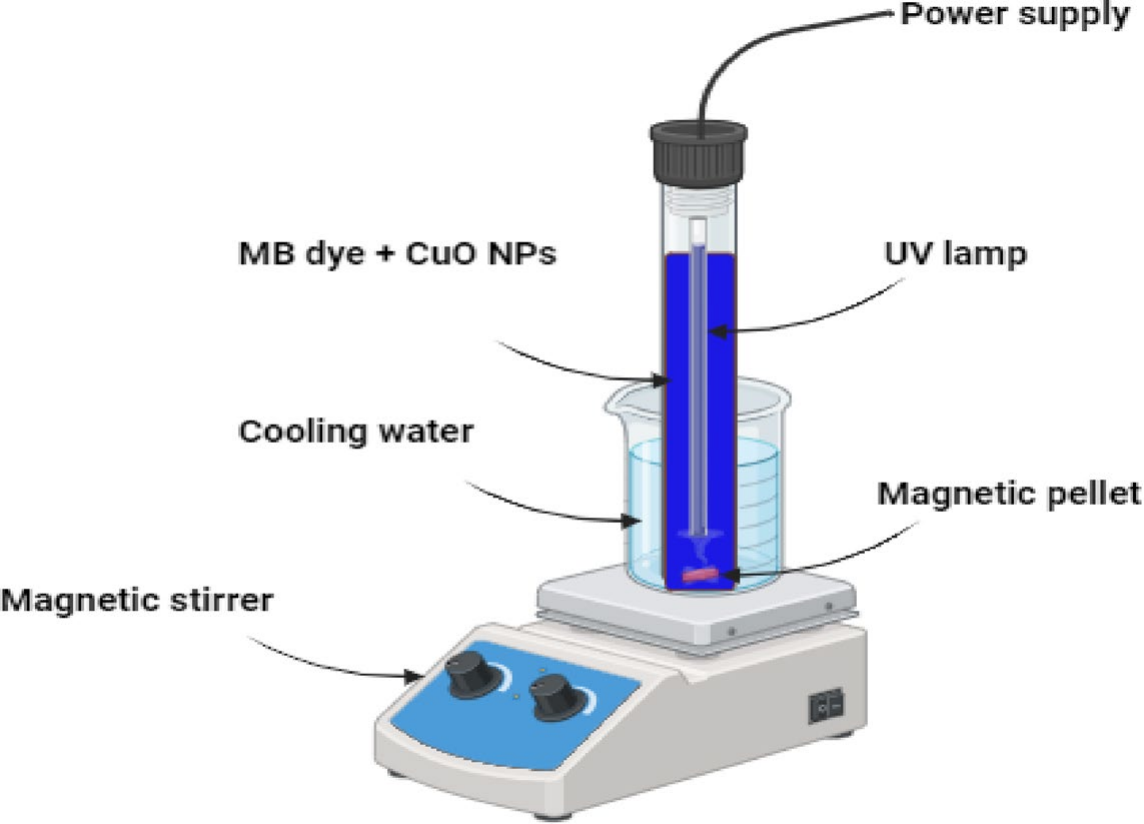
Schematic figure of the photocatalytic setup.

MB degradation was accomplished using photocatalysis employing a UV lamp and CuO-NPs. The current UV reactor in use is a cylindrical glass tank with a volume of 100 ml. Figure 1 depicts that it has a diameter of 3 cm and a length of 27 cm. The reactor’s surface is covered with a thin layer of aluminum foil. The photoreactor was filled with 50 ml of contaminated solutions. The ultraviolet light source used was the Philips TUV 11WG11 T5, a widely available UV-C lamp. With an output of 11 watts with ultraviolet radiation of 30.000 W/cm^2^, this high-pressure mercury lamp emits light with an average wavelength of 254 nm. The photoreactor is completely immersed in the contaminated solutions while keeping a temperature of around 15 °C through the utilization of a cold-water bath.

Firstly, inject pollutant MB dye and nanocatalyst into the glass cylindrical reactor. Next, administer UV irradiation. At regular intervals, withdraw a 1 ml suspension of the MB solution from the UV reactor using a syringe. Apply centrifugal force to the suspension for a duration of 20 min and quantify the amount of light absorbed at a wavelength of 664 nm using a spectrophotometer. The photodecomposition efficiency (Removal %) was calculated from the following equation:

1 $$\:\mathbf{Removal }\:\% =\left(\frac{\varvec{C}0-\varvec{C}\varvec{t}}{\varvec{C}0}\right){*}100$$

Where C_o_ refers to the initial concentration of MB in mg/L, while C_t_ reflects the concentration at a specific time (t). An investigation was conducted to examine the operational parameters of photocatalytic degradation, which included the initial concentrations of contaminants and the pH level.

### Antimicrobial activity and minimal inhibitory concentration of CuO-NPs

The antibacterial efficacy of the synthesized CuO-NPs was assessed using the agar-disc diffusion method^24^ against Gram-negative bacteria (*Escherichia coli ATCC 25922)* and Gram-positive bacteria (*Staphylococcus aureus ATCC 25923)*. The efficacy of the CuO-NPs was assessed by comparing it to conventional antibiotic discs containing Ciprofloxacin (CIP) at a concentration of 5 µg/ml and a diameter of 6.0 mm.

The minimum inhibitory concentrations (MIC) of the tested substance, which exhibited the strongest antibacterial activity, were determined using the serial dilutions method on the Luria-Bertani (LB) medium^25^. These assessments utilized a negative control, which was the medium broth; a positive control, which was the pathogenic bacteria being researched; and the medium broth and synthesized CuO-NPs, commencing with a concentration of 20.0 µg/ml. MIC was determined over a 24-hour incubation period at a temperature of 36.0 ± 1.0 ˚C ^26^.

### Insect rearing

*Culex pipiens* was reared in the entomology laboratory of the Nuclear Research Center, EAEA, Cairo, Egypt, and maintained under laboratory conditions of 25 ± 2 ˚C and 60–70% (R.H.) and 12:12 h (Light: Dark)^27^.

### Larval bioassay

The effects of different concentrations of CuO-NPs (20, 40, 60, 80, 100, and 120 mg/L) were tested on freshly hatched 3rd and 4th instar larvae. Twenty-five larvae were introduced into glass jars containing 200 ml of dechlorinated tap water that had been combined with predetermined quantities of CuO-NPs. Each concentration was tested in three duplicates, and a control group was kept in dechlorinated tap water. The mortality percentage was calculated after a period of 5 days, and the LC_25_, LC_50_, and LC_90_ values for larvae in the 3rd and 4th instar stages were determined using probit analysis^28^. The data were corrected for control mortality using Abbott’s formula^29^.

### Statistical analysis

The data were analyzed using one-way analysis of variance (ANOVA) and were presented as means ± standard error (S.E.) using IBM Corp.‘s SPSS software, version 25 (New York). The significance level was established at a P-value of less than 0.05. The lethal concentrations (LC) and their corresponding 95% confidence intervals were computed using Ldp line Software, version 1.0 that calculate probit analyses according to Finney (1971)^30^.

##  Results and discussion

### Characterization of CuO-NPs

#### X-ray diffraction analysis

**Fig. 2:**
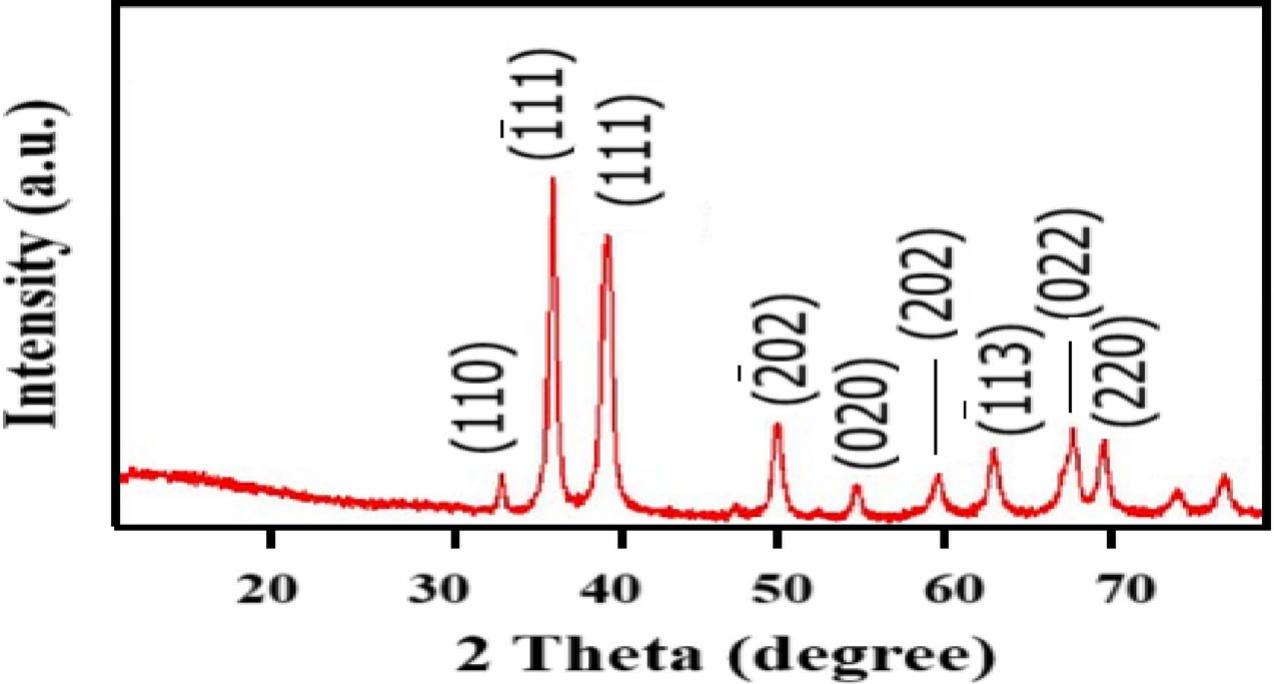
XRD patterns of CuO-NPs annealed at temperature 600 °C.

The presence of CuO-NPs was verified using XRD analysis, as illustrated in Fig. 2. The presence of monoclinic crystallite can be inferred from the detection of planes. A matching diffractogram in the 2*θ* range 10–80 with a Bragg’s reflection and the notable 2*θ* values observed were 33.15, 36.10, 39.78, 49.87, 54.72, 58.98, 62.79, 67.23, and 69.82, which correspond to the (110), (111^−^), (111), (202^−^), (020), (202), (113^−^), (022) and (220) planes, respectively in the XRD analysis. These planes do not exhibit any peaks, suggesting the absence of impurities such as Cu_2_O and Cu (OH)_2_. These are nearly identical to the ones found in the JCPDS (card no. DB 01-080-1917), as depicted in Fig. 2. The results are comparable to the data that has been reported^31–33^. Our findings are consistent with previously reported results. For instance, Cao et al. (2021) observed similar diffraction peaks of monoclinic CuO-NPs indexed to JCPDS card no. 01-080-1917, confirming the absence of Cu₂O or Cu(OH)₂ impurities^34^.

#### Scanning electron microscopic and high-resolution transmission electron microscope analysis

**Fig. 3:**
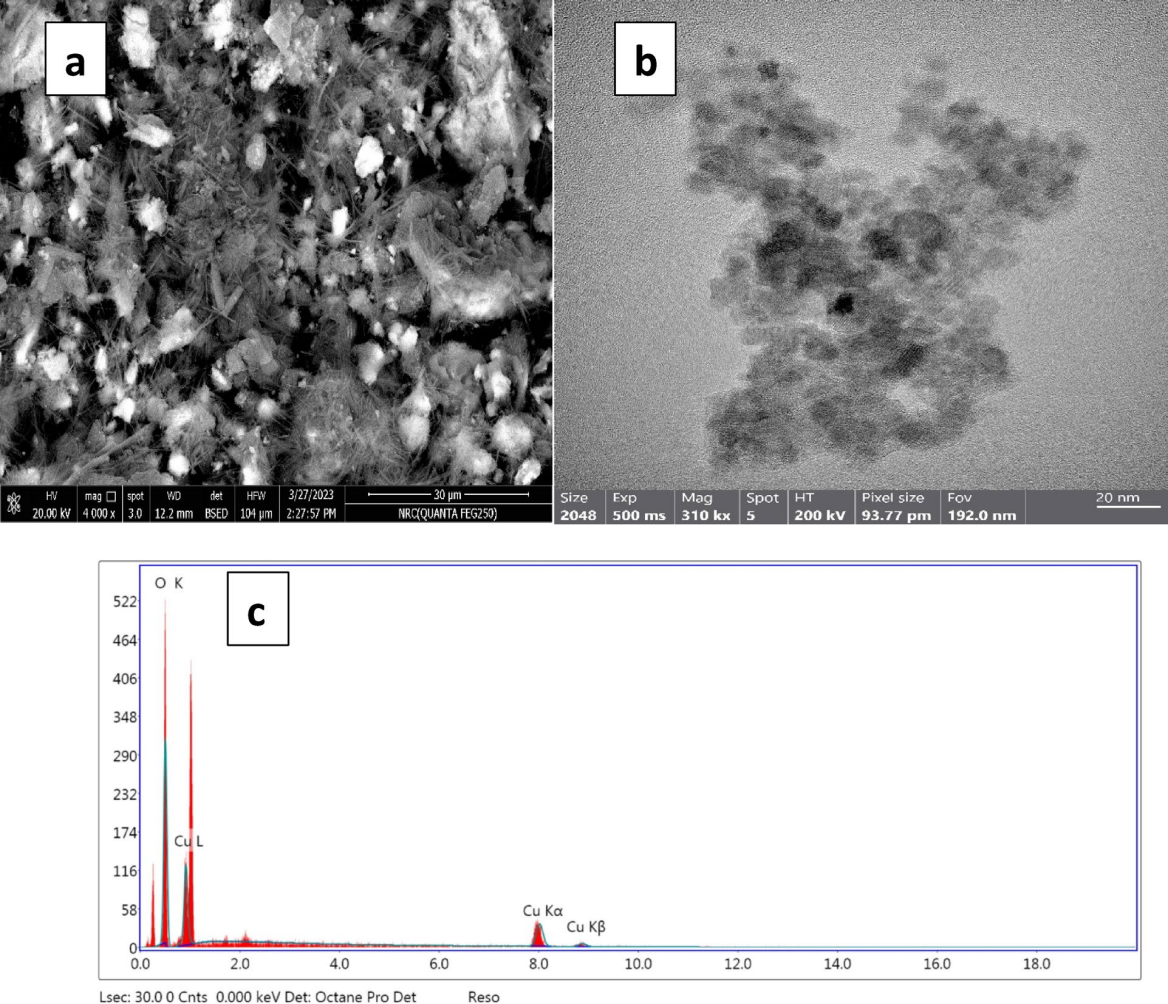
(**a**) SEM image of CuO-NPs, (**b**) HRTEM image of CuO-NPs and (**c**) EDX analysis of synthesized CuO-NPs.

The scanning electron microscopy (SEM) and energy-dispersive X-ray spectroscopy (EDX) images in Fig. 3a & c illustrated the CuO-NPs obtained by annealing at a temperature of 600 °C. The SEM image of CuO-NPs indicated the existence of evenly distributed spherical particles with a clear and consistent crystalline structure. Furthermore, there was a heightened tendency for clusters. The SEM image verifies the existence of a regular polyhedron shape for the CuO-NPs. The proliferation of the closely packed spherical arrangement was clearly observed. In specific regions, the larger nanoparticles were surrounded by smaller nanoparticles. Observations and records were made of identical SEM images depicting CuO-NPs^35^. The size of the synthesized CuO-NPs was assessed using HRTEM, as depicted in Fig. 3b. HRTEM revealed that the nanoparticles had a mainly spherical morphology. HRTEM imaging revealed a particle size distribution ranging from 8 to 22 nm, which aligns with the previously reported size range of CuO-NPs in other studies^36,37^. These morphological observations are in good agreement with previous reports. Luna et al. reported that CuO-NPs obtained by annealing at 600 °C exhibited well-dispersed spherical particles with a tendency to agglomerate, as well as polyhedral features surrounded by smaller nanoparticles^38^. Similarly, Mishra et al. confirmed that CuO-NPs present mainly spherical morphology with particle sizes in the range of 8–25 nm, which is consistent with our HRTEM analysis^39^. These findings indicate that the synthesized CuO-NPs in this study display structural and size characteristics comparable to those widely reported in the literature.

#### Fourier transform infra-red analysis

**Fig. 4:**
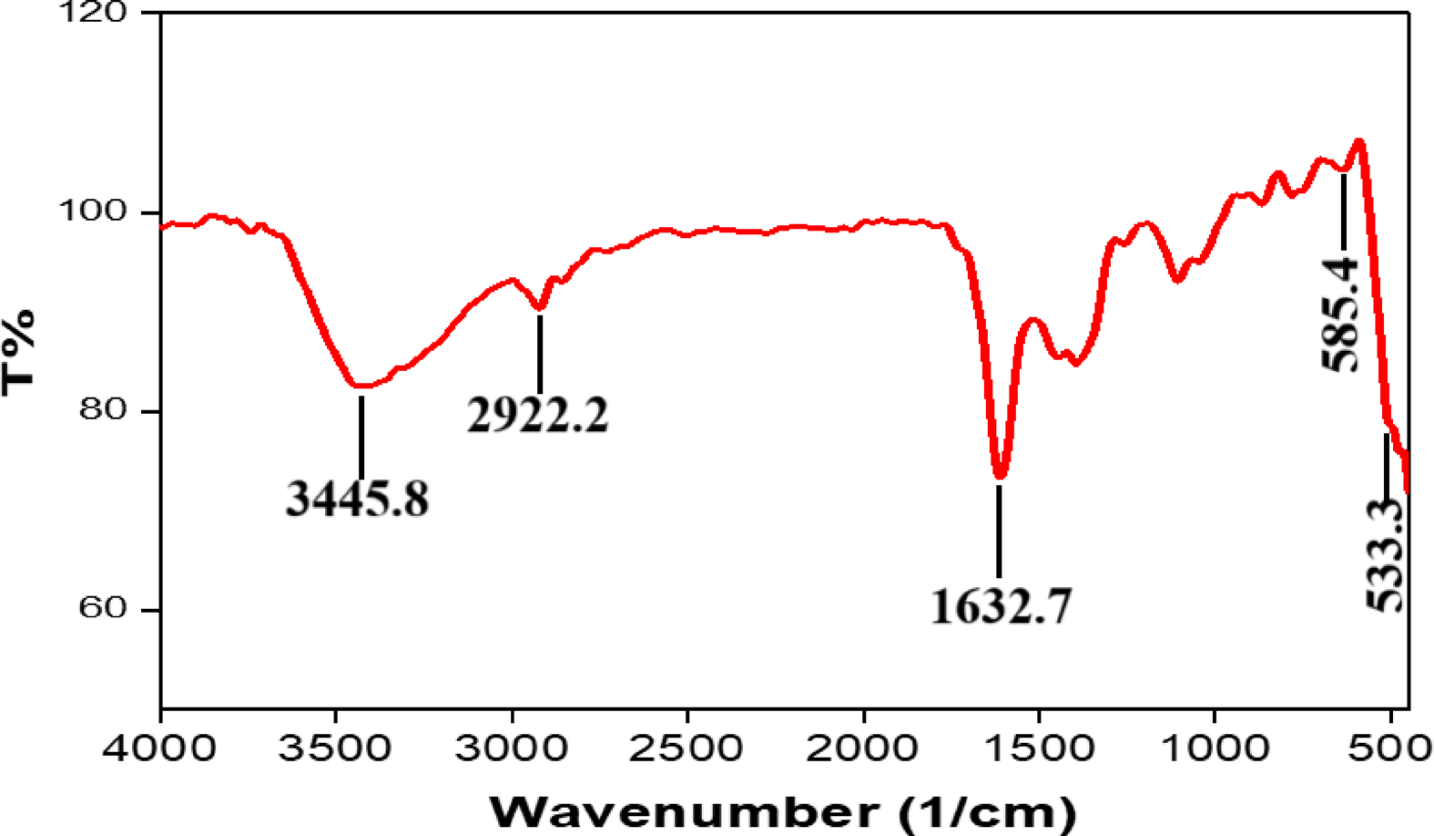
FTIR spectrum of the synthesized CuO-NPs.

Figure 4 exhibits the Fourier Transform Infra-red (FTIR) spectra of CuO-NPs that have been subjected to annealing at a temperature of 600 °C. The existence of adsorbed water molecules led to a broad absorption peak at around 3445.89 cm^− 1^. Nano crystalline materials possess the capacity to absorb moisture as a result of their elevated surface-to-volume ratio. Prior research has recorded the existence of a similar highest point at 3434 cm^− 1^ in the FTIR spectra of CuO-NPs^35^. The signal observed at 2922.21 cm^− 1^ is a result of the stretching of the -C-H bond, which is specifically assigned to the alkyl group^40^. The spectral range from 2700 to 3750 cm^− 1^ is designated as the OH-stretching area^41^. The observed peaks at 1632.77 may correspond to the symmetrical stretching of Cu-O bonds^42^. The vibrational modes of CuO-NPs are shown by two infrared absorption peaks within the region of 500–700 cm^− 1^. The peaks were detected at wavelengths of 533.33 cm^− 1^ and 585.41 cm^− 1^, respectively. The peak observed at 533.33 cm^− 1^ may be attributed to the stretching of Cu-O bonds^43^. The little deviation in the vibrational modes is linked to the concurrent alteration in the surface area of the produced CuO-NPs^44^. The occurrence of peaks at 533.33 cm^− 1^ and 585.41 cm^− 1^ indicates the formation of the CuO-NPs. These two peaks indicate the presence of the monoclinic phase. The lack of any additional infrared active modes between the 500–700 cm^− 1^ range conclusively refutes the existence of CuO. The FTIR spectra of CuO-NPs display two distinct peaks at 525 cm^− 1^ and 580 cm^− 1^, which closely align with our own observations^45^. Hence, the metal-oxygen frequencies detected for CuO-NPs closely correspond to the values reported in the literature. Our FTIR spectrum of CuO-NPs (annealed at 600 °C) shows a broad OH band at ~ 3446 cm⁻¹ due to adsorbed surface water, a weak C–H stretching signal near 2922 cm⁻¹, and a band around 1633 cm⁻¹ commonly attributed to H–O–H bending. Two strong Cu–O vibrations at ~ 533 and ~ 585 cm⁻¹ confirm monoclinic CuO; the absence of additional IR-active modes between 500 and 700 cm⁻¹ further rules out Cu₂O, consistent with prior reports^38,45^.

### Photocatalytic degradation of MB dye using CuO-NPs

**Fig. 5:**
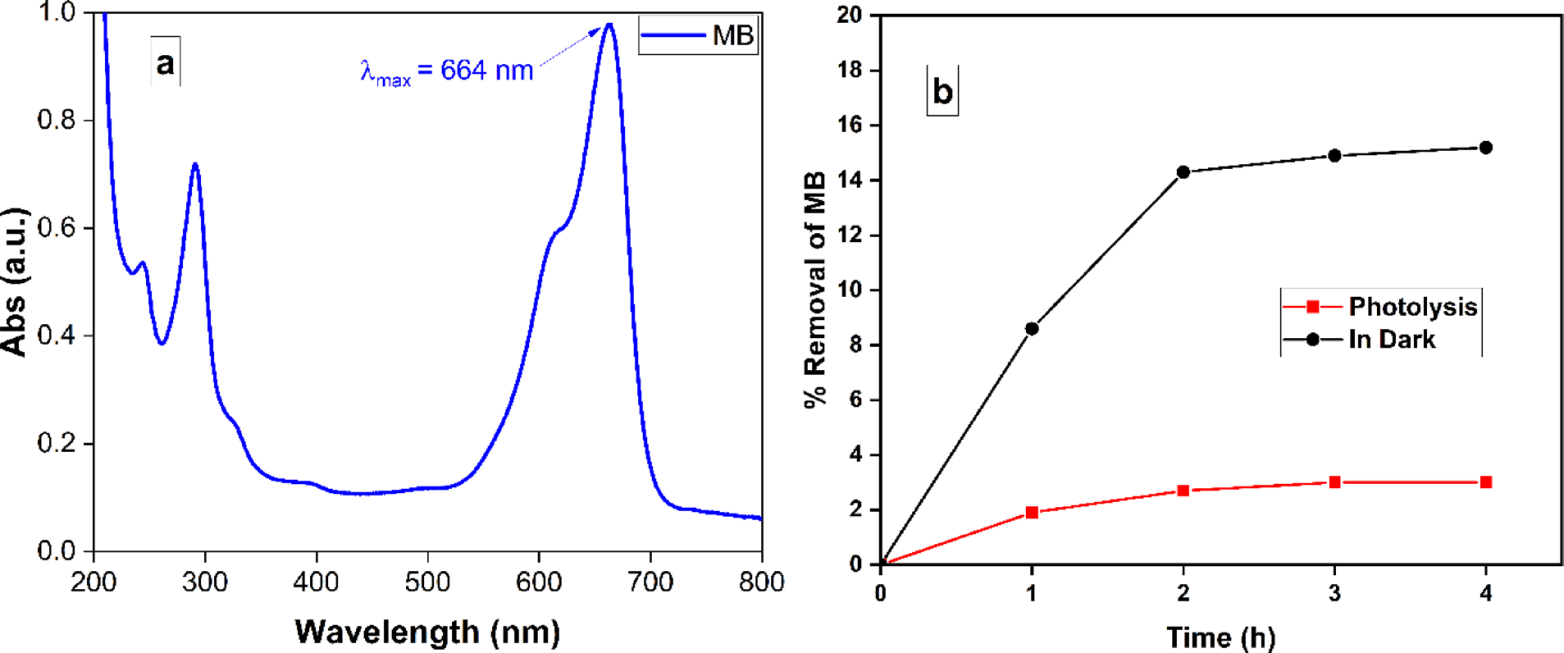
(**a**) Absorbance reduction in the spectrum of MB with time due to photocatalysis, (**b**) % removal of photolysis (red line) and adsorption activity in the dark of CuO-NPs (black line).

The removal of MB was tracked using spectrophotometry at the absorbance peak of the MB dye, which is λmax = 664 nm, as depicted in Fig. 5a^17^. Figure 5b depicated that the breakdown of MB caused by photolysis after 4 h was only 7.0%, while the removal due to adsorption in the absence of light was 14.9% during the same conducted time. The improved photocatalytic performance can be attributed to the influence of the created metal-semiconductor heterojunction of the nanoparticles, which enhances the separation of charges and the absorption of incoming light.

#### Effect of pH on removal of MB

**Fig. 6:**
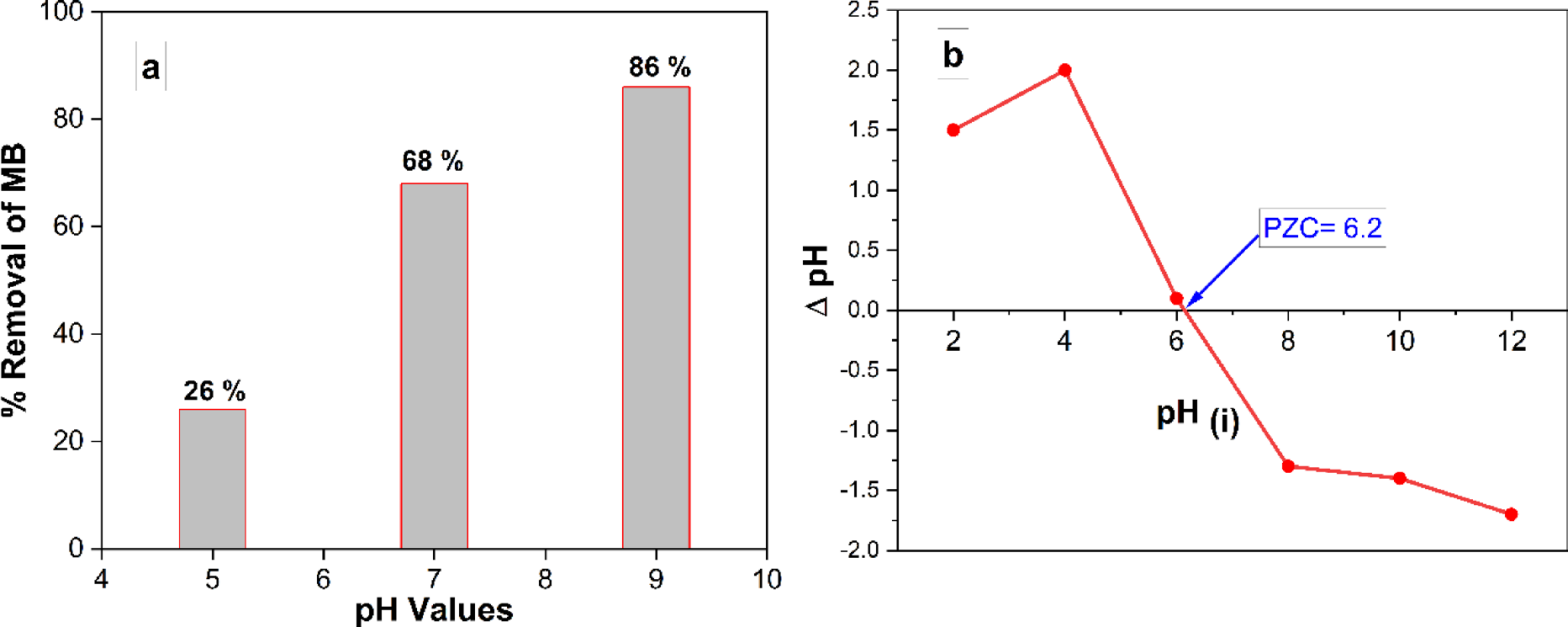
(**a**) Variation of MB removal (%) with time at different solution pH (5.0, 7.0, and 9.0) (10 mg of CuO-NPs in 50 ml of 10 mg/L MB at 25 °C), (**b**) Point of zero charge (PZC) of CuO-NPs at different pH values.

When carrying out removal investigations, it is crucial to take into account the pH dependence of the solution. An investigation was conducted to assess the effect of various initial pH values on the MB solution. The investigation involved using 10 mg of CuO-NPs, 50 ml of a 10 mg/L MB solution, and maintaining a temperature of 25 °C. The investigation was conducted over a period of 90 min. Figure 6a depicted a graph that shows the percentage of MB elimination over time at three different solution pH levels (5.0, 7.0, and 9.0). The pH of 9.0 resulted in the greatest elimination of MB at equilibrium. To determine the point of zero charge (PZC) of the CuO-NPs, 0.01 g of CuO-NPs was added to a 50 ml solution containing 0.01 moles of NaCl. The pH of the solutions was adjusted by adding either HCl or NaOH in order to obtain pH values of 2, 4, 6, 8, 10, and 12. The specimens were stirred at a velocity of 200 revolutions per minute for a period of 48 h. pH values of the solutions were determined subsequent to the magnetic separation of CuO-NPs.

PZC was determined by graphing the initial pH versus the pH change (Initial pH - Final pH). Figure 6a presented the displayed results. According to Fig. 6b, PZC is the pH value at which there is no discernible difference between the final and initial pH readings. Here, the PZC was found to have a pH value of 6.2. The photocatalyst CuO-NPs exhibits a positive surface charge when the pH is lower than the pH of PZC and a negative surface charge when the pH is higher than the pH of the PZC. Moreover, when the pH of the solution aligns with the pH at PZC, the surface charge of the photocatalyst becomes neutral, leading to a minimal electrostatic force between the photocatalyst surface and ions, specifically MB ions^46^. The pH value at which the CuO-NPs reached their PZC was found to be 6.2. The discovery explains the observed maximum degradation of MB through photocatalysis at pH 9.0, as depicted in Fig. 6a. Thus, now, the CuO-NPs possess a negative net surface charge, leading to an electrostatic attraction with the positive charge of MB. This attraction intensifies the process of photocatalytic degradation of MB. The degradation of MB through photocatalysis began to decrease at a pH of 5.0 due to the positive overall surface charge of the CuO-NPs. The presence of a positive charge resulted in repulsive forces between the positive charge of MB and the overall positive surface charge of the CuO-NPs at a pH of 5.0.

#### Effect of initial concentration of MB and nanocatalyst dose on photocatalytic reaction

**Fig. 7:**
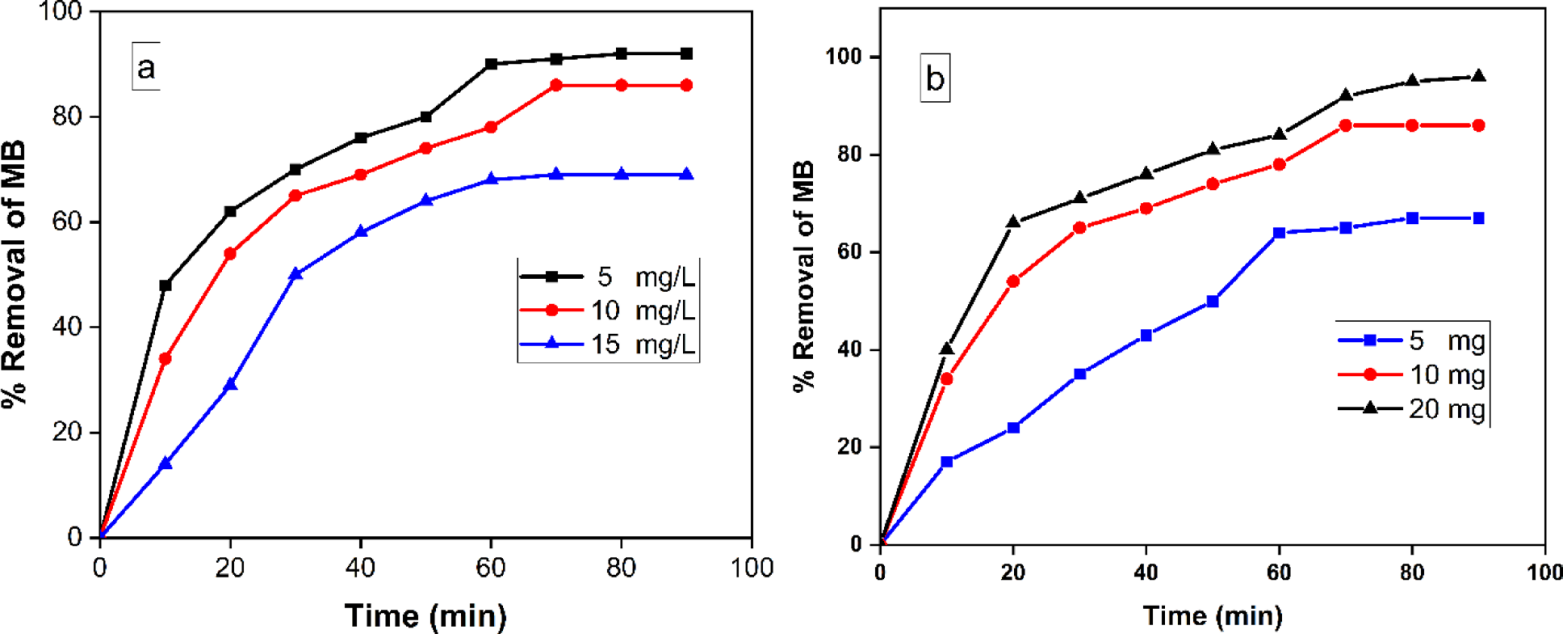
(**a**) Variation of percent removal as a function of contact time at different initial MB concentrations (5–15 mg/L) at pH 9.0 and 10.0 mg CuO-NPs, (**b**) Effect of the photocatalyst dose on the removal efficiency of MB (50 ml MB solution (10 mg/L), Temp. = 25 ˚C and pH 9.0).

The effect of ionic strength on MB was investigated by altering its initial concentration while keeping all other reaction parameters unchanged. The initial MB concentration plays a vital role in the elimination process. Figure 7a depicted the correlation between the percentage of elimination and the duration of contact for different beginning concentrations of MB (5.0, 10.0, and 15.0 mg). Figure 7a depicted the declining effectiveness of the CuO-NPs generated in reducing the concentration of MB at different initial concentrations. The results suggest that the efficiency of degradation diminishes as the concentration of MB rises. Nevertheless, the nanocomposite effectively eliminates MB even at elevated initial concentrations under UV light exposure.

An investigation was conducted to assess the effect of varying dosages of NPs on the efficiency of removing MB under UV light. The quantity of the photocatalyst employed was altered within the range of 5–20 mg, while the concentration of MB remained constant at 10 mg/L. This is depicted in Fig. 7b. The results indicated that the removal efficiency showed a notable improvement as the dosage of the photocatalyst increased from 5 to 20 mg. The increase in removal efficacy seen when the amount of photocatalyst in the reaction is increased can be attributed to the corresponding increase in the accessible active area or active sites of the photocatalyst in relation to the volume ratio of the MB solution^47^.

#### Kinetic studies

**Fig. 8:**
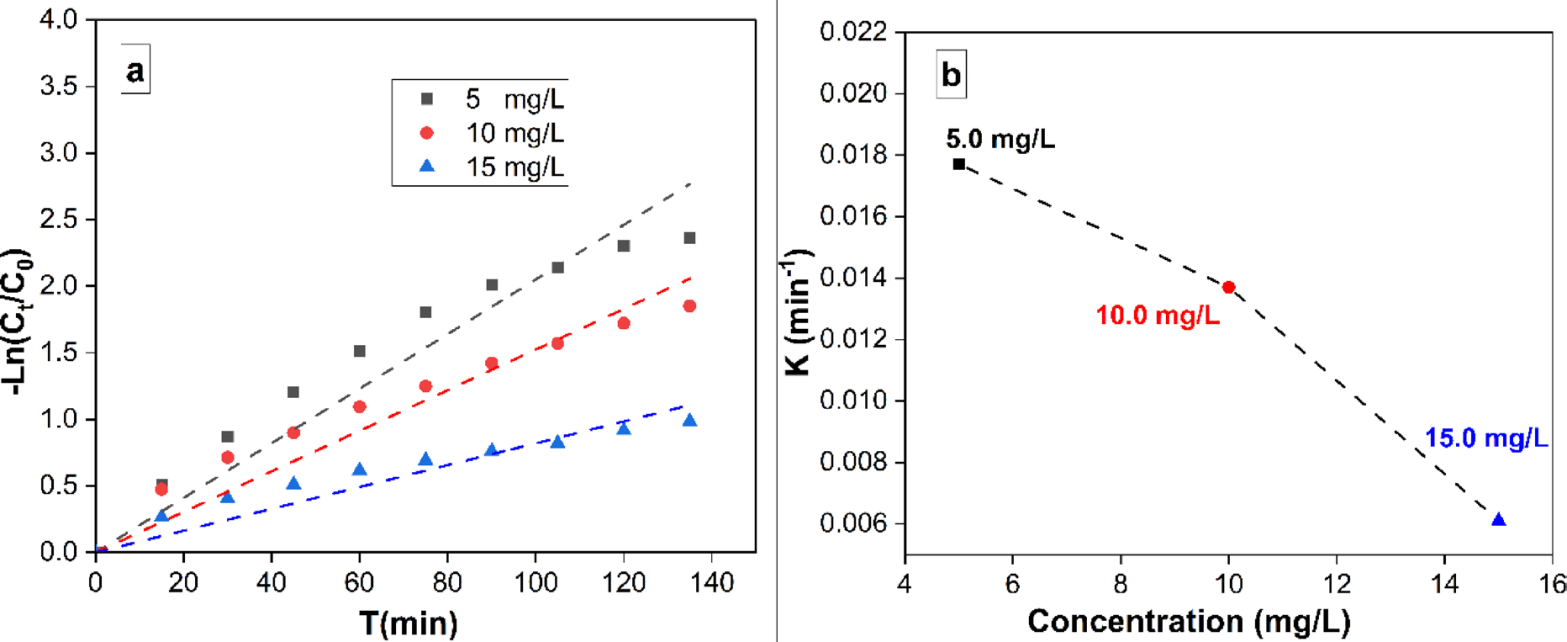
(**a**) A linear fit, pseudo-first-order model data are reported in kinetic form for MB degradation under UV-light irradiation with MB concentrations of 5, 10, and 15 mg/L. (**b**) Shows a relation of apparent pseudo-first-order model constants vs. initial concentration of MB.

The MB removal rate was computed using the following equation:

2 $$\:-ln\:\left(\frac{Ct}{C0}\right)\:=\:-\:Kt\:$$

where C_t_ represents the residual concentration of MB, whereas C_o_ represents the initial concentration of MB. The time taken for removal is represented as t, while the removal rate constant is marked as k. Figure 8a depicts the relationship between the natural logarithm of the ratio of C_t_ to C_o_ and the variable t. The results indicate that the rate of the elimination procedure conformed to a pseudo-first-order model. An augmentation in the initial concentration of MB resulted in a proportional augmentation in the apparent pseudo-first-order rate constants. The dependence of reaction rate constants on MB concentration matches earlier literature, as indicated in Figure 8b^48^.

#### Reuse and recycling

**Fig. 9:**
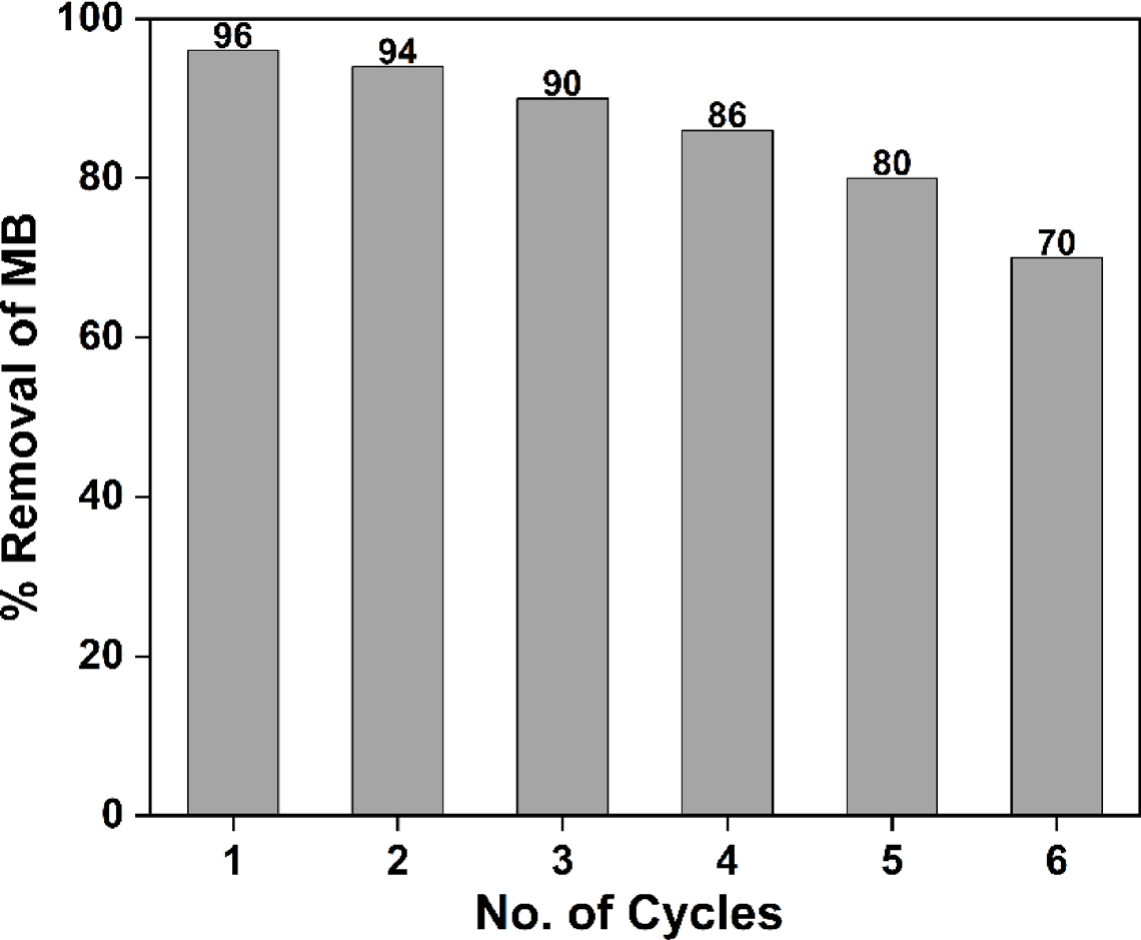
Recyclability of CuO-NPs for MB degradation under UV light irradiation.

The waste management requires the extraction and reuse of photocatalysts employed in environmental remediation^49^. Consequently, the CuO-NPs sample demonstrated enhanced photocatalytic degradation efficiency and cost-effectiveness. Photocatalyst stability and long-term photocatalytic activity are both critical features. Reuse stability of a photocatalyst is essential to its industrial applications. The photocatalyst was collected by centrifugation, cleaned thrice with deionized water then let dry in an oven for eight hours at 60 °C before being used in the next cycle. Further research was carried out, as indicated in Fig. 9, to examine the reusability of CuO-NPs in the photocatalytic reduction of MB dye under UV light irradiation. The photocatalytic reusability was conducted in the same manner as for the assessment of photocatalytic activity previously described. The photocatalytic activity of CuO-NPs reduced to 70% after 6 cycles.

#### Mechanism of MB photocatalysis

The photodegradation mechanism, which is sensitive to changes in pH, may be involved in the possible process. In this process, hydroxyl radicals attack and oxidize via the valence band’s positive holes and are reduced by the conduction band’s electrons. One possible mechanism by which photocatalytic degradation could take place is the creation of electron-hole pairs on the photocatalyst’s surface upon exposure to ultraviolet light^50,51^.

Both hydroxyl radicals and degradation products can be generated by the oxidative potential of holes, depending on whether they react with -OH groups or oxidize the reactive MB. Here is a brief overview of the processes involved in the photocatalytic degradation of MB: (3–6).

3 $${\text{CuO-NPs}} + {\text{ hv}}~ \to {\text{CuO-NPs }}\left( {{\text{e}}^{ - } _{{{\text{CB}}}} + {\text{ h}}^{ + } _{{{\text{VB}}}} } \right)$$

4 $${\text{h}}^{ + } _{{{\text{VB}}}} + {\text{ CuO-NPs}}~ \to {\text{CuO-NPs}}^{ + } \left( {{\text{Oxidation of the compound}}} \right)$$

Or

5 $${\text{h}}^{ + } _{{{\text{VB}}}} + {\text{ OH}}^{ - } \to {\text{OH}}^{.}$$

6 $${\text{OH}}^{.} + {\text{ MB}} \to ~\left( {{\text{Degradation products}}} \right)$$

**Fig. 10:**
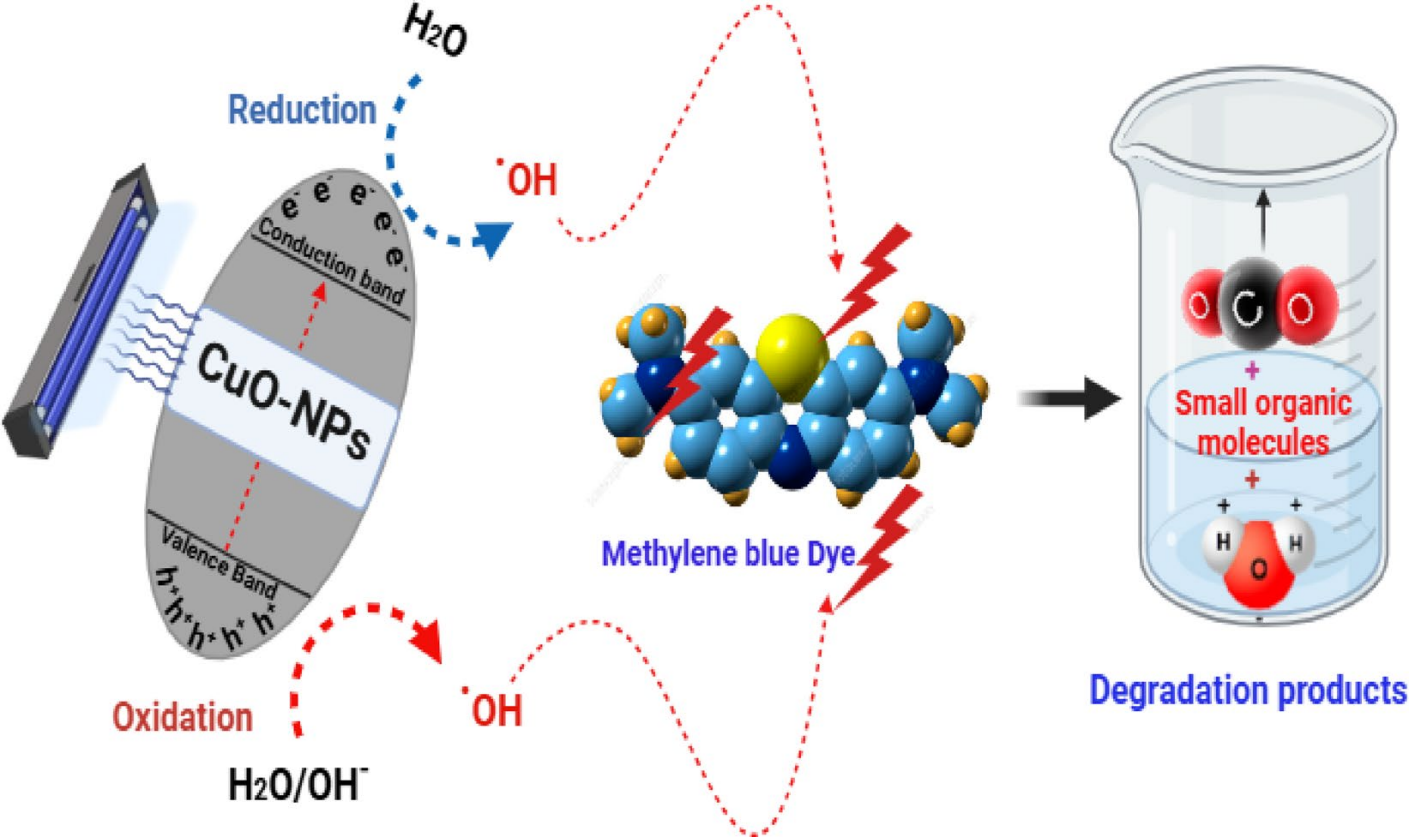
The possible photocatalytic reaction mechanism for MB photodegradation by CuO-NPs.

Figure 10 illustrates the suggested mechanism that explains how the CuO-NPs and MB interact. The absorption of photons by CuO-NPs in the presence of visible light can initiate redox reactions by generating charge carriers. Consequently, the created free radicals, including OH^·^ and O2^·−^, can aid in the decomposition of MB, leading to the formation of less complex organic molecules.

**Fig. 11:**
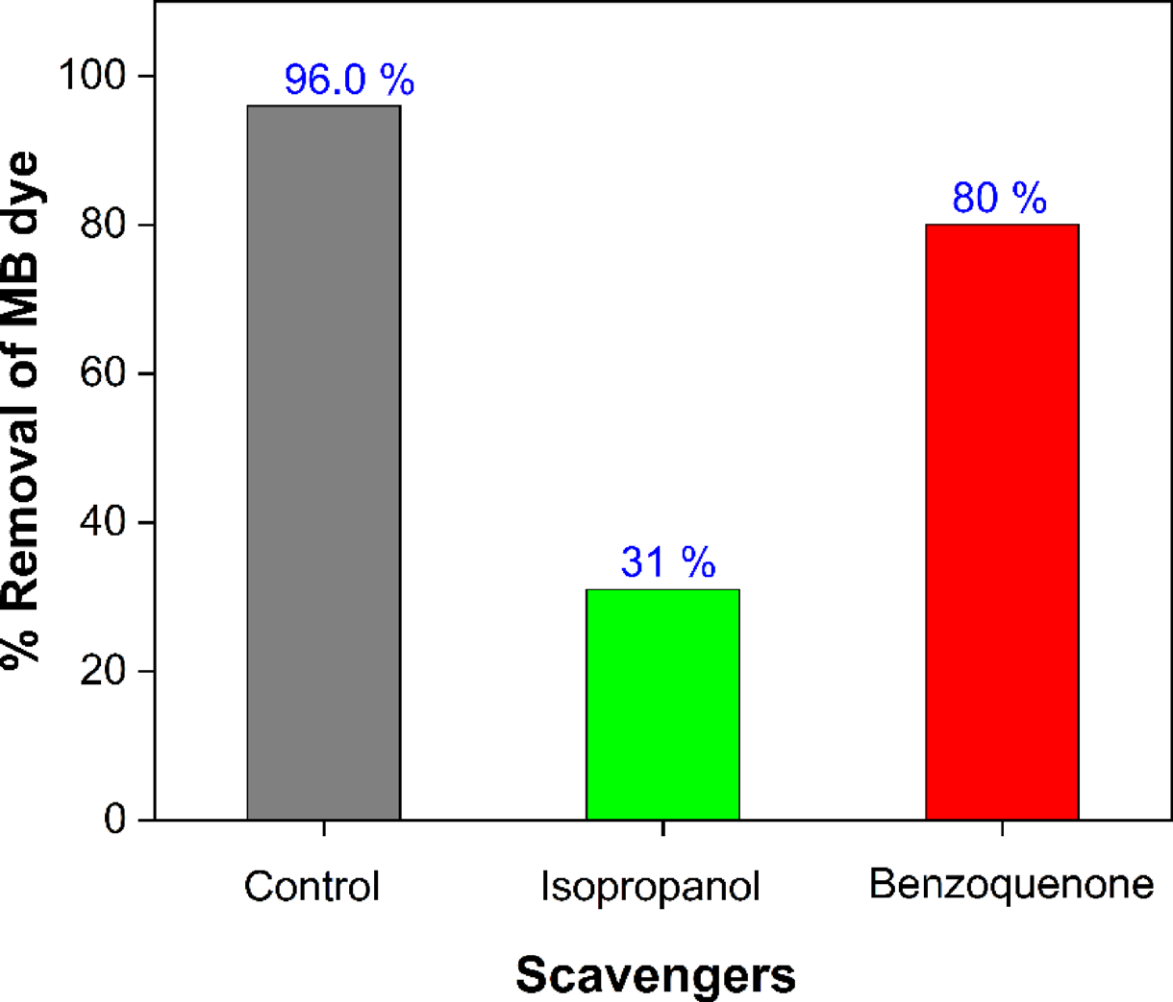
Effect of scavengers on MB photodegradation by CuO-NPs.

The most active species were determined by analyzing how scavengers affect the photocatalytic reduction process. The isopropanol and benzoquinone were used to capture OH^·^ and − O_2_^·^, respectively^46^. CuO nanocomposite’s photocatalytic degradation of MB under UV irradiation is illustrated in Fig. 11, which illustrates the efficiency of this process in the absence and presence of scavengers at a concentration of 5 ppm. Efficiency decreased from 96% to approximately 80% and 31% in the presence of benzoquinone and isopropanol, respectively. The removal of MB by the CuO-NPs was reduced to (31%) when isopropanol was added, indicating that the OH^•^ radical was the primary species responsible for MB degradation. In addition, the degradation rate was reduced by 80% by the addition of benzoquinone, which indicates that the − O_2_^·^ radical played a significant role in the degradation of MB^52^. The potential of the synthesized CuO-NPs as a promising candidate for sustainable environmental application can be seen by comparing the photodegradation results with the existing literature as listed in Table 1.

**Table 1:** A comparative study of the photocatalytic properties of CuO nanostructures for the degradation of various organic dyes, including the present study.

Photocatalyst	Synthesis method	Particle size (nm)	Light source	Dye/pollutant	Time (min)	Degradation rate	Ref.
Redispersible CuO NPs	Ethylene glycol method	~ 92 nm	UV	MB	75	99.6%	^53^
CuO NPs	Green synthesis	5–92 nm	–	CR	120	95%	^54^
				Ciprofloxacin	210	80%	
CuO nanosheets	Hydrothermal	~ 40–60 nm	Sunlight	MB	70	99	^55^
				RhB		90	
CuO/MgAl-LDH composite	Co-precipitation	~ 20–40 nm	Visible	MB	80	99.2%	^56^
SDS-CuO NPs	Co-precipitation	~ 27–95 ± 5 nm	UV	MB	90	94%	^57^
CuO via *Cocos nucifera*	Green synthesis	60 nm	UV–Vis	MG	100	94%	^58^
				EBT		70%	
CuO-NPs	Chemical precipitation	8–22 nm	UV	MB	90	96%	**This study**

### Antimicrobial efficacy of the synthesized CuO-NPs

#### Antimicrobial activity and MIC of CuO-NPs

Synthesized CuO-NPs showed a strong antibacterial action against the tested bacteria, according to the disc agar distribution method, which was utilized as a screening step. In Table 2, we can see that the synthesized CuO-NPs exhibited strong antibacterial activity against *S. aureus* (ZOI of 23 mm) and *E. coli* (ZOI of 17.0 mm) according to the in vitro zone of inhibition (ZOI) test. The produced CuO-NPs were more effective against Gram-positive bacteria than Gram-negative ones. Lipopolysaccharide, lipid, and peptidoglycan are the main components of Gram-negative bacteria’s cell walls. On the other hand, Gram-positive bacteria have peptidoglycans that are extremely compressed in their cell walls^59^. The results against *S. aureus* and *E. coli* for the MIC of CuO-NPs were 0.625 and 1.25 µg/mL, respectively, as shown in Table 2.

**Table 2:** Antimicrobial activity of CuO-NPs against gram-positive and gram-negative bacteria measured as ZOI (mm) and MIC (µg/ml).

Pathogenic bacteria	ZOI of CuO-NPs (20.0 µg/ml) (mm)	MIC of CuO-NPs (µg/ml)	CIP
*S. aureus*	23.0 ± 0.524	0.625	22.0 ± 0.652
*E. coli*	17.0 ± 0.784	1.25	27.0 ± 0.457

#### Mechanism of antibacterial action of the synthesized CuO-NPs

**Fig. 12:**
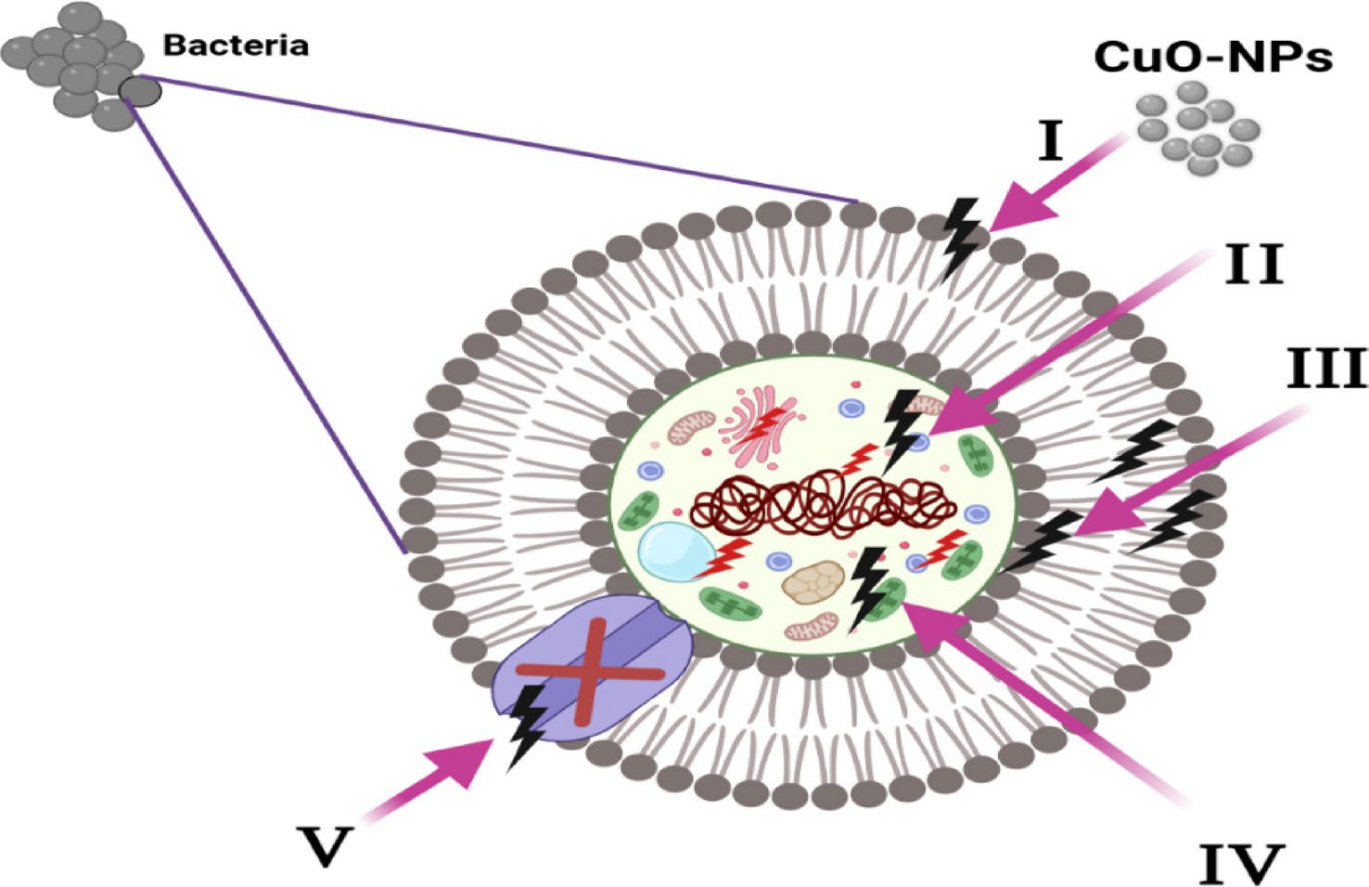
Diagrammatic depiction of the four primary mechanisms by which the synthesized CuO-NPs exhibit antibacterial activity: (**I**) CuO-NPs bind to and encase the surface of the microbial cell, disrupting membrane function and changing transport activity. (**II**) Once within the microbial cells, the CuO-NPs interact with several biomolecules and organelles, including ribosomes, chromosomes, DNA, and mesosomes, which in turn affects the corresponding cellular machinery. (**III**) The produced CuO-NPs cause extracellular ROS to form and grow, which in turn damages cells. (**IV**) CuO-NPs trigger cell death by influencing the cellular signaling pathway. (**V**) Lastly, CuO-NPs prevent ions from entering and leaving the cells of microbes.

The antibacterial activity of the CuO-NPs that were synthesized was explained. Once produced, CuO-NPs begin to work by encasing and adhering to the surface of the microbial cells, which causes the cell membrane to rupture and changes the transport capacity^60^. The next step is for the NPs to disperse throughout the microbial cell, which triggers the division of all important organelles and intracellular components like DNA and plasmids. The generation of reactive oxygen species (ROS) leads to oxidative stress, which in turn causes cellular damage. Figure 12 depicated that the nanocomposite blocks ion transport to and from the microbial cells. Table 3 listed some metal oxides nanoparticles and their application as antibacterial agent.

**Table 3:** Previous studies of some metal oxide nanoparticles for antibacterial applications.

Nanoparticle type	Synthesis method	Particle size (nm)	Antibacterial applications	Ref.
Copper oxide NPs (CuO)	Green synthesis Using *Aerva javanica* Leaf Extract	15–23	Antibacterial activity against bacterial pathogens of *E. coli*., *P. aeruginosa*., *A. baumannii*., *S. aureus.*	^61^
Copper oxide NPs (CuO)	Green synthesis using *Cardiospermumhalicacabum* extract	10 ± 2	Antibacterial activity against Gram-positive organisms such as *Bacillus subtilis*,* Staphylococcus aureus*, and Methicillin Resistant *Staphylococcus aureus.*	^62^
Copper oxide NPs (CuO)	Green synthesis using copper (Cu) tolerant bacterial isolate *Serratia* sp. ZTB29 strain	20–40	Antimicrobial activity against *Xanthomonas* Sp. and *E. coli*	^63^
Copper oxide NPs (CuO)	Green synthesis using ethanolic extracts of *Azadirachta indica* and *Simmondsia chinensis*	10.7–30.9	Antibacterial activity against clinical isolates, including methicillin-resistant *S. aureus* (MRSA), *E.coli*, *P.aeruginosa*, *Acinetobacter* spp., *Klebsiella pneumoniae*, and *Stenotrophomonas maltophilia*	^64^
Copper oxide NPs (CuO)	*Green synthesis using Calotropis procera* leaf extract	20–80	Anti-fungal and anti-bacterial activities	^65^
Copper oxide NPs (CuO)	Green synthesis using *Zingiber officinale* Rhizome Extract	4.35	Antibacterial activity against *S.aureus* ATCC 25,926 and *E.coli* ATCC25922	^66^
Copper oxide NPs (CuO)	Green synthesis Using pomegranate (*Punica granatum*) extracts	< 100	Antibacterial activity against Gram-negative bacteria	^67^
Copper oxide NPs (CuO)	Green synthesis using Aloe Vera Miller leaf extract	21.3 ± 2.1	Achieving an MIC of 62.5 µg/mL against both *E. coli* and *S. aureus* and > 99.9% bacterial kill within 4 h of colloidal manifest.	^68^
Copper oxide NPs (CuO)	Green synthesis using Peel of Pomegranate extract.	36.99–55.17	antibacterial activity of Cu (NO_3_)_2_ nanoparticle against Salmonella (ATCC^®^9270TM)	^69^
Copper oxide NPs (CuO)	Green synthesis using ginger (*Zingiber officinale*, ZO) and garlic (*Allium sativum*, AS) extracts.	< 50	Antibacterial potential of extract doped CuO NPs against pathogenic *E. coli*.	^70^
Magnesium oxide NPs (MgO)	Green synthesis using the metabolites secreted by brown algae, *Cystoseira crinita*,	3–18	promising activities against Gram-positive bacteria, Gram-negative bacteria, and *Candida albicans* with MIC values ranging between 12.5 and 50 µg mL^− 1^	^71^
Silver NPs (AgNPs)	Green synthesis using *Trichoderma viride* filtrate	19.6 ± 6.1	antimicrobial activity compared to OCH alone, with low MIC values against *P. aeruginosa*, *Candida albicans*, *Aspergillus brasiliensis*, and *Staphylococcus aureus* MRSA	^72^

### Toxicity of CuO-NPs on *Cx. pipiens*

**Fig. 13:**
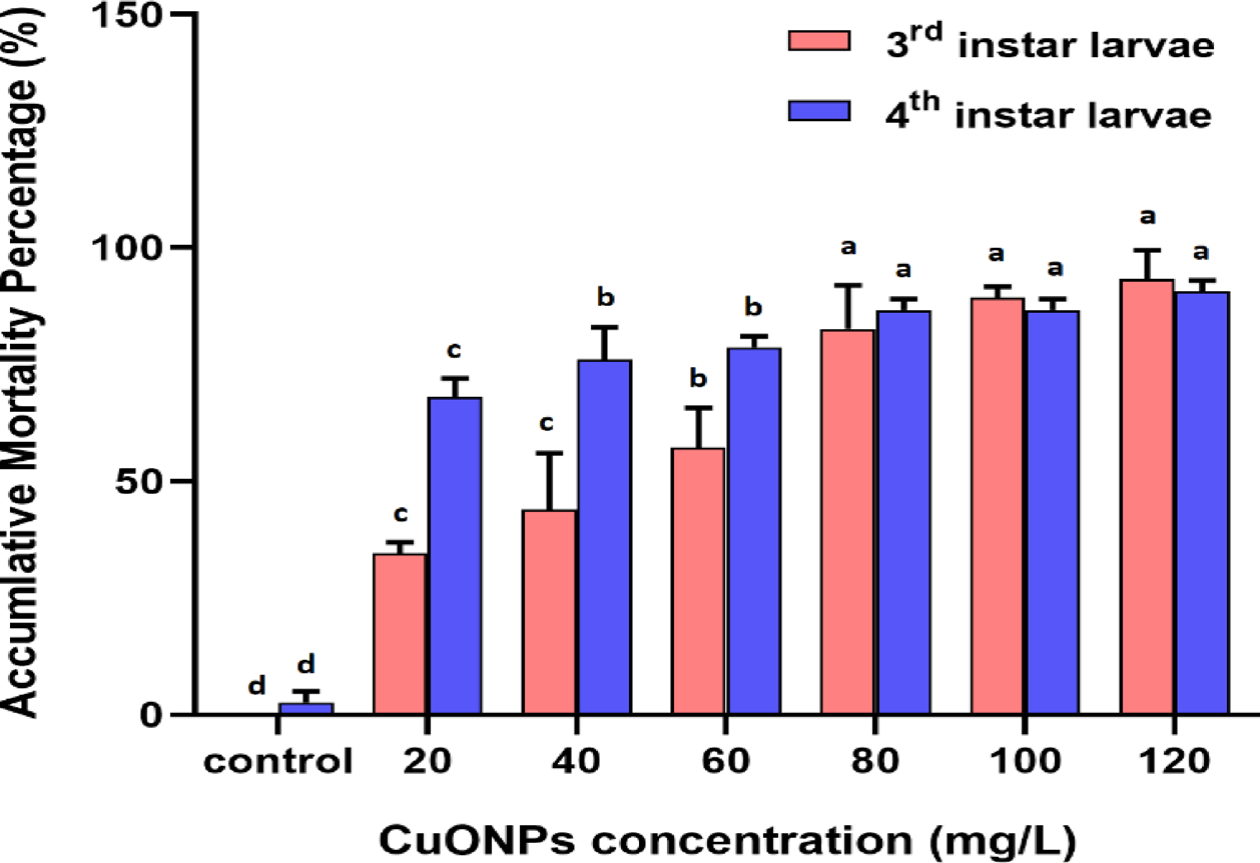
Accumulative mortality percentage of 3rd and 4th instar larvae of *Cx. pipiens* treated with CuO-NPs. After 5 treatment days Bars with the same color and different letters denote significant differences at *P* < 0.05.

**Fig. 14:**
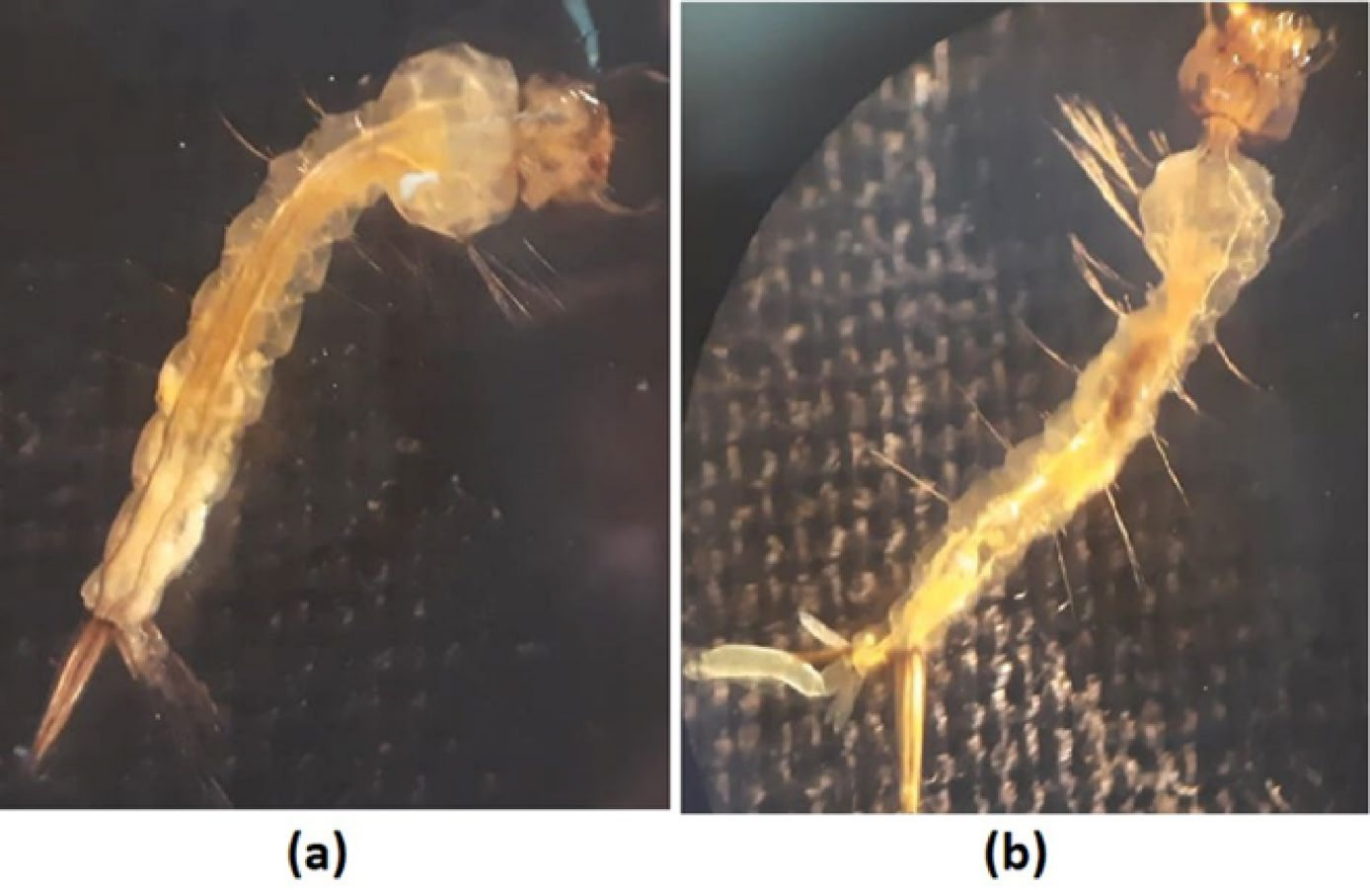
Light microscopy graph of 3rd instar larvae of *Cx. pipiens*. (**a**) Control larvae. (**b**) 5 days of treated larvae with CuO-NP, X = 40.

The death rate was assessed at 24, 48, 72, 96, and 120 h. Results showed that compared to the control group, the cumulative mortality percentage in third and fourth instar *Culex pipiens* larvae increased significantly when CuO-NPs concentrations grew. See Figure 13 after the 5-day treated period. The probit analysis also showed that the LC_50_ values for the third instar larvae were 37.61 mg/L and 8.31 mg/L for the fourth instar larvae. Figure 14 showed the morphological study of untreated and treated 3rd instar larvae with an LC_50_ of CuO-NPs; the body appeared with normal head, thorax and abdomen regions Figure 14a. However, after 5 days of CuO-NP treatment, the larvae lost their movement and became inactive with unclear segmented bodies and disintegrated midguts Fig 14b.

**Table 4:** Effect of CuO-NPs on the 3rd and 4th larval instars of *Cx. pipiens* after 5 days.

CuO-NPs (mg/L)	LC_25_ (mg/L) (95% confidence limits)	LC_50_ (mg/L) (95% confidence limits)	LC_90_ (mg/L) (95% confidence limits)	Slope	χ^2^ value	*R* value
III-instar	20.05 (7.47–23.15)	37.61 (21.21–48.37)	124.22 (104.82–287.85)	2.47 ± 0.25	13.87	0.94
IV-instar	1.87 (0.08–5.71)	8.31 (1.43–15.97)	140.96 (94.33–369.59)	1.04 ± 0.25	1.09	0.96

According to Table 4, the fourth instar larvae showed a significant level of vulnerability. According to Thandapani et al.^73^, green-synthesized CuO-NPs demonstrated a notable ability to kill *Cx. quinquefasciatus* larvae. Earlier Muthamil Selvan et al.^74^ showed the toxicity of green-synthesized CuO-NPs against *Aedes aegypti* at LC_50_ 4.2 mg/L. Later on, CuO-NPs revealed a toxic effect on *Cx. pipiens* and *Musca domestica* larvae^75^. Subsequently, biosynthesized CuO-NPs recorded toxicity against *Tribolium castaneum* at LC_50_ 37 mg/L after 5 days of treatment^76^.

One possible explanation for CuO-NPs’ larvicidal effects is that they penetrate the insect exoskeleton via the epithelial cells^77^. Also, the lymphatic capillaries are a common pathway for nanoparticles, which can cause oxidative stress and, in the end, cell damage^78^. Similarly, *Fadl et al.* found that the translocated NPs followed the same path to the hemolymph and ROS production in the cells; therefore our findings are in line with theirs^79^. Precious recent studies were a significant increase in glutathione S transferase activity & inhibition in AChE activity, down-regulation in AChE gene expression, midgut epithelial cells lysis, and destruction in microvilli and nuclei upon larval treatment with SeNPs especially the fourth instar over the third one found by Awad et al. 2025 ^80^. This provides evidence for the neurotoxicity and genotoxicity of NPs on the *Cx. pipiens* larvae. Hematological abnormalities in the cells lining the midgut were also readily apparent. Furthermore, CuO-NPs can bind to DNA phosphorus and protein -SH groups when ingested or enter cells through the intracellular space. Denaturation of proteins and interference with gene expression are possible outcomes^75^. Table 5 listed some previous studies of nanoparticles for larvicidal activity.

**Table 5:** A comparative study of the larvicidal efficacy of some metal and metal oxides nanoparticles.

Nanoparticle type	Synthesis method	Particle size (nm)	Target species (Larvicidal)	Larvicidal efficacy (LC50 / Mortality % & duration)	Refs.
Selenium Nanoparticles (SeNPs)	Green synthesis using *Trichoderma viride* filtrate	25.4–80.6	*Culex pipiens* (4th instar larvae)	LC_50_ = 39.2 mg/L (24 h)	^80^
iron oxide nanoparticles	Green synthesis using *aqueous leaf extract of Grevillea robusta*	41.9	*Aedes aegypti*	LC_50_ = 259.07 mg/L for the 2nd instar larvae LC_50_ = 238.05 mg/L for the 4th instar larvae (12–48 h)	^81^
			*Anopheles stephensi*	LC_50_ = 297.96 mg/L for the 2nd instar larvae LC_50_ = 292.72 05 mg/L for the 4th instar larvae (12–48 h)	
Silver Nanoparticles (AgNPs)	Green synthesis using *Trichoderma viride* filtrate	14.28–30.58	*Culex pipiens* (4th instar larvae)	LC_50_ = 52 mg/L (24 h)	^80^
Selenium Nanoparticles (SeNPs)	Green synthesis using *Cupressus sempervirens* extract + microwave irradiation	11–55	*Culex pipiens* complex (3rd instar larvae)	LC_50_ = 28.25 mg/L (9 days)	^79^
	Green synthesis using *Cupressus sempervirens* extract + gamma irradiation	21–75	*Culex pipiens* complex (3rd instar larvae)	LC_50_ = 31.28 mg/L (9 days)	^79^
Copper Oxide Nanoparticles (CuONPs)	Green synthesis using *Achillea fragrantissima* extract	4.61–6.97	*Culex pipiens* (3rd instar larvae)	LC_50_ = 0.67 mg/L (48 h)	^82^
Olive Cake Hydrolysate-Silver Nanoparticles (OCH-AgNPs)	Green synthesis using *Pseudomonas fluorescens*-mediated olive cake hydrolysate	19.6 ± 6.1	*Culex pipiens* (2nd & 3rd instar larvae)	LC₅₀ = 0.40 mg/L (24 h)	^72^
Alumina Nanoparticles (Al₂O₃)	Auto-combustion with glucose fuel at pH 7, calcined at 800 °C (Al-G7-800)	3.9	*Culex pipiens* (1st, 2nd, 3rd, 4th instar larvae & pupal stage)	100% mortality (1st instar, 200 mg/L)(24 h)	^83^
Silica Nanoparticles (silica NPs)	Hydrophilic silica NPs (purchased)	8.39 ± 2.35	*Culex pipiens* (3rd instar larvae)	100% mortality (400 mg/L) (72 h)	^84^
Zinc Oxide Nanoparticles (ZnO NPs)	Purchased	10–30	*Culex pipiens* (1st instar larvae)	LC_20_ = 0.24 g/L (72 h treatment, chronic effects)	^85^
Copper oxide Nanoparticles (CuO-NPs)	Precipitation method	8–25	*Culex pipiens*	LC_50_ 3rd instar = 37.61 mg /L LC _50_ 4th instar = 8.31 mg /L (5days)	**This Study**

## Conclusion

This study highlights the multifunctional potential of synthesized CuO-NPs, showcasing their effectiveness across environmental and biological applications. The CuO-NPs were thoroughly characterized using XRD, SEM, HRTEM, and FTIR, confirming their structural integrity and nanoscale morphology. Their photocatalytic performance, particularly in degrading methylene blue under UV irradiation, demonstrated promising efficiency influenced by factors such as pH, dye concentration, and catalyst dosage. Kinetic modeling further confirmed that the degradation process follows a pseudo-first-order reaction. In addition to their environmental application, CuO-NPs exhibited significant antimicrobial activity against both Gram-positive and Gram-negative bacteria, suggesting potential for use in infection control and antimicrobial formulations. Moreover, the larvicidal assay against *Culex pipiens* revealed a strong dose-dependent toxic effect, with LC₅₀ values indicating their promise as eco-friendly alternatives to conventional insecticides. The current study utilizes conventional chemical synthesis methods for producing CuO nanoparticles, which may involve toxic reagents and energy-intensive procedures. This raises concerns about environmental safety, cost, and biocompatibility, limiting the applicability of the nanoparticles in eco-sensitive or biomedical environments. Without green synthesis, the resulting CuO-NPs might retain chemical residues or exhibit properties that are less biocompatible or more toxic to non-target organisms, including humans and beneficial microbes. Chemical synthesis may face limitations in scalability due to high production costs, stringent safety requirements, and waste generation, which could hinder industrial or large-scale environmental applications. Future work should explore the biosynthesis of CuO-NPs using plant extracts, fungi, bacteria, or agro-waste. These methods offer a more sustainable and eco-friendlier alternative, often producing nanoparticles with enhanced stability, functionalization, and biocompatibility. In the context of future society, such multifunctional nanomaterials could play a pivotal role in addressing challenges related to pollution, infectious diseases, and vector-borne illnesses through sustainable and scalable technologies.

## Data Availability

The data used to support the findings of this study are available from the corresponding author upon request.
